# Well-Being and Functioning at Work Following Thefts and Robberies: A Comparative Study

**DOI:** 10.3389/fpsyg.2018.00168

**Published:** 2018-02-20

**Authors:** Ilaria Setti, Peter G. van der Velden, Valentina Sommovigo, Maria S. Ferretti, Gabriele Giorgi, Deirdre O'Shea, Piergiorgio Argentero

**Affiliations:** ^1^Unit of Applied Psychology, Department of Brain and Behavioural Sciences, University of Pavia, Pavia, Italy; ^2^INTERVICT, Tilburg University, Tilburg, Netherlands; ^3^Kemmy Business School, University of Limerick, Limerick, Ireland; ^4^Department of Psychology, European University of Rome, Rome, Italy

**Keywords:** violence at work, post-traumatic stress disorder (PTSD), psycho-somatic well-being, trauma-related coping self-efficacy (CSE), job satisfaction

## Abstract

Thefts and robberies may be traumatizing experiences for employees. The aim of this study is to explore to what extent experiencing robberies and/or thefts at work affect workers' mental health, coping-self-efficacy, social support seeking, workload and job satisfaction. Drawing on Conservation of Resources theory, this research contributes to our understanding of the psychological sequelae of robbery and theft for employees working in small businesses. The few studies on the effects of robberies and thefts in the past have predominantly focused on bank employees. A sample of Italian tobacconists and jewelers completed an anonymous self-report questionnaire examining the experience of robbery and/or theft, social support seeking (Coping Orientation to Problem Experienced scale, COPE-IV), psycho-somatic well-being (General Health Questionnaire, GHQ-12), job satisfaction (a single item). Victims of thefts and/or robberies reported their PTSD symptoms (Impact of Event- Revised 6, IES-R-6) and trauma-related coping self-efficacy (Coping Self-Efficacy scale, CSE-7), based on the last event (*N* = 319). Descriptive analyses, ANOVA, ANCOVA and multiple regressions analyses have been carried out. The results indicated that victims of thefts and robberies experienced greater workload, higher psycho-physical complaints and greater tendency to seek social support in comparison with their non-affected counterparts. They additionally experienced more post-traumatic symptomatology and perceived lower coping self-efficacy, when compared to those who experienced thefts “only.” Multiple regression analyses revealed that CSE was positively related to job satisfaction, although the presence of psycho-physical symptoms was the main predictor of job satisfaction among both non-affected and affected employees. PTSD was not an independent predictor of job satisfaction. In sum, robberies and/or thefts exposure undermines differently workers' well-being.

## Introduction

Robberies and thefts represent a serious threat to workplace safety. For instance, in Italy the numbers of robberies and thefts in 2015 among retailers were 5,337 and 102,041 respectively (Istat, 2015). In fact, Italy had the twelfth highest robbery rates out of 71 countries in 2006 (Aebi et al., 2010; European Institute for Crime Prevention and Control International Statistics on Crime Justice, 2011). To date, only a few studies have focused on the psychological impact of these types of events on employees working in small, independently owned businesses (Casteel et al., 2008; Söndergaard, 2008; Belleville et al., 2012), with previous research on this topic predominantly focusing on the banking context (Van der Velden et al., 1992; Kamphuis and Emmelkamp, 1998; Hansen and Elklit, 2011, 2013; Hansen et al., 2012, 2014; Armour and Hansen, 2015; Christiansen and Hansen, 2015; Giorgi et al., 2015a,b; Mucci et al., 2015). The present research focused on jewelers and tobacconists because their job characteristics (e.g., customer-facing, working alone or in a small team, handling valuables and selling items of value) may potentially increase workers' risk for theft and robbery-related violence. The main purpose of this study was to investigate how experiencing robberies and/or thefts at work may affect workers' psychological well-being and job satisfaction. Indeed, previous research has widely demonstrated that being exposed to traumatizing experiences—such as robberies—may negatively affect individuals' mental health (Van der Velden et al., 1992; Hansen et al., 2014) with resulting effects on work-related aspects, such as job satisfaction (Giorgi et al., 2015b).

Both robberies and thefts are considered property crimes, perpetrated by “anyone who gains possession of another person's movable goods, stealing them from the owner with a view to drawing profit for themselves or others” (Article 624, 628 C.P.; Lattanzi, 2010). The key distinguishing factor between a theft from a robbery is that the latter involves the use of “personal violence or threats” of force (Article 628 C.P.; Lattanzi, 2010). Since such experiences are consistent with the conditions described by criterion A for PTSD as defined by the Diagnostic and Statistical Manual of Mental Disorder (DSM-V), thefts and robberies may be considered fully-fledged potentially traumatic events (PTEs).

Previous studies have shown that a robbery may be a traumatizing experience for workers, which may initiate the development of or increase existing mental health problems, such as psychological distress, major depression, acute stress disorder, and post-traumatic stress symptoms (Van der Velden et al., 1992; Kamphuis and Emmelkamp, 1998; Hansen and Elklit, 2014a,b; Hansen et al., 2014). This, in turn, may lead victims to experience reduced overall job satisfaction (Giorgi et al., 2015b), decreased work productivity (Zatzick et al., 2008), and increased demand for medical and mental healthcare services (Van der Velden et al., 1992; Mucci et al., 2015).

Experiencing a robbery or theft constitutes a threat to one's working conditions, which are one of four resources identified by Conservation of Resources (COR) theory (i.e., objects, conditions, personal characteristics, energies; Hobfoll, 1989). Following the exposure to a theft or robbery, workers experience a loss in their resources with regard to their working conditions such as their personal safety at work and the perception of a secure working environment. If workers (continue to) suffer from resource loss without being able to compensate through resource replenishment by employing other resources to offset the loss, they are at increased risk for mental health problems and PTSD symptoms. Furthermore, employing resources for coping can be stressful too (Hobfoll, 1989). If the resources expended in coping are greater than the resulting benefits, then the outcome of coping is likely to be negative (Hobfoll, 1989). Thus, if workers expend energy trying to cope with the aftermath of the theft or robbery, but are unsuccessful in doing so, they may further exacerbate their losses. In this situation, the likelihood for post-traumatic complaints and continued health impairment are more likely. In a situation of continued resource depletion, work demands may be perceived as more effortful due to an individual's reduced coping capacity. COR theory suggests that stress is not the result of an imbalance between objective demands and resource capacity, but rather the perception of these factors (Hobfoll, 1989). Little research to date has considered the effect of robberies and thefts on the regular everyday demands of one's job, such as perceived workload. However, it is likely that if one's resources are already depleted due to a traumatic event than a worker's capacity to deal with everyday work demands is reduced and thus, workload may be perceived to be higher. We investigate this aspect in the present study.

In the context of resource loss, or threat of loss, the harnessing of other types of resources becomes important (e.g., resource replacement; Hobfoll, 1989) to meet the demands related to the recovery, restore lost resources or reduce the negative impact. Social support seeking is an active coping strategy (Schaefer and Moos, 1998; Norberg et al., 2006; Taylor and Stanton, 2007) that may facilitate this process following robberies and thefts. Social resources may foster victim's resilience and recovery in the aftermath of a trauma because it may have a positive impact on the victims' perceptions of social support competence (Lepore, 2001) and the belief about their ability to cope with the stressor (Schwarzer and Knoll, 2007) by decreasing feelings of isolation (Ozer et al., 2003). Furthermore, when constructively provided, social support may encourage successful adaptation by helping victims to re-appraise the traumatic event and thus, engage in more effective coping strategies (Schaefer and Moos, 1998; Prati and Pietrantoni, 2009). In sum, receiving social support may reduce the emotional burden of robberies and thefts (Thoits, 2011).

Since robberies are different from thefts (because by definition the level of violence is more severe in a robbery), we expected that thefts and robberies would have differential impacts on workers' well-being. To examine these expectations, normally we would distinguish the following three groups of victimized workers: (1) workers who experienced thefts only; (2) workers who were victims of robberies only; (3) workers who were targeted of both thefts and robberies. However, at the beginning of our study we became aware that the majority of (if not all) robbed workers were also confronted with thefts (or vice versa). For this reason, the subgroup including those who experienced robberies only was eliminated and we focused on the comparison between the other two affected groups.

**Hypothesis (1)** Workers employed in jewelers and tobacco shops who were affected by both robberies and thefts will experience higher levels of psycho-somatic complaints and higher perceived workload, and they will seek more social support compared to non-affected workers.

PTEs like robberies and thefts may be accompanied by more specific post-traumatic symptoms such as re-experiencing the event, avoiding event-related feelings and places, event-related anger and fear in the short, medium or long term. Some victims may develop a mental disorder, such as PTSD (DSM-5; American psychiatric association, 2013). The development of psycho-physical problems and post-traumatic stress symptoms may depend on the severity of trauma exposure and how it is subjectively experienced (Tsai et al., 2012). Furthermore, individuals exposed to multiple traumatic events may experience higher levels of post-traumatic symptoms and more severe impairment of mental health compared to those exposed to single events (Schwartz et al., 2005; Wahlström et al., 2008; Wisnivesky et al., 2011; Yuan et al., 2013; Karam et al., 2014). Taken together, these findings suggest that being exposed to both robberies and thefts may exacerbate the detrimental outcomes over and above being exposed to thefts only.

**Hypothesis (2)** Workers who were exposed to both robberies and thefts will be more likely to experience PTSD symptomatology than colleagues who were victims of thefts only.

As stated by COR theory (Hobfoll, 1989), personal resources are beneficial because they help individuals to deal with stressors. In the context of trauma exposure, trauma-related coping self-efficacy (CSE) is an important personal resource, which has been found to protect individuals from psychological distress (Benka et al., 2014) and is positively associated with psychological well-being (Benight et al., 2001; Lambert et al., 2012). CSE refers to the perceived ability to effectively manage both personal functioning and external recovery demands handled in the aftermath of a traumatic event (Benight and Bandura, 2004). CSE impacts the perceived stressfulness of traumatic events through various mechanisms. First, CSE influences the extent to which an event is perceived as threatening through an appraisal of the balance between personal coping abilities, environmental demands and potentially harmful characteristics of the event (Bandura, 1997). Secondly, CSE perceptions affect both the motivation to utilize and sustain effective coping strategies and the choice of adopted strategies, thereby influencing the perception of the event as stressful over time (Benight et al., 1999; Kraaij et al., 2002; Benight and Bandura, 2004). Thirdly, since CSE regards the belief in one's own ability to relieve stress reactions, it plays an important role because it helps victims to perceive stressors as less distressing (Kent, 1987; Kent and Gibbons, 1987). CSE perceptions are continuously adapting over time on the basis of individuals' perceptions of their own effectiveness in dealing with emotional and psychological consequences of the trauma. The level of stress perceived during or immediately after the traumatic event seems to have an impact on both short-term symptoms and the ability to overcome the event (Bosmans and van der Velden, 2015; Bosmans et al., 2015a). This suggests that the perceived seriousness of the event could affect CSE levels, so that traumatic events perceived as more serious may lead individuals to experience lower CSE perceptions.

**Hypothesis (3)** Workers who were exposed to both robberies and thefts will report lower CSE levels than those who were victims of thefts only.

The negative association between psychological well-being and job satisfaction has been widely demonstrated across several types of professional populations (Faragher et al., 2005; Alexopoulos et al., 2014). A longitudinal study by Giorgi et al. (2015b) showed that robberies had long-term effects on victims' psychological distress, which in turn may lead to reduced overall job satisfaction. In addition, a meta-analysis by Bowling et al. (2015) revealed that workload was significantly and negatively associated with job satisfaction, although this association was relatively weak. Consequently, we expected that workers who reported greater psycho-physical complaints and higher perceived workload would be less satisfied with their job.

Furthermore, coping self-efficacy perceptions have been shown to be particularly important for job satisfaction (Judge et al., 2001; Schyns and von Collani, 2002). A strong relationship was found between feeling generally competent and being satisfied with one's job (Judge et al., 2001; Law and Guo, 2016). More specifically, previous studies found that occupational self-efficacy predicts job satisfaction (Schyns and von Collani, 2002; Paggi and Jopp, 2015). However, to the best of our knowledge, no studies investigating the relationship between the more specific construct of trauma-related CSE and job satisfaction have been carried out to date. Feeling more confident in one's ability to cope with trauma should protect employees against detrimental psychological consequences and, in turn, contribute to the maintenance of job satisfaction levels following a theft or robbery. Some studies have found relationships between PTSD and job dissatisfaction (North et al., 2002; Paunović and Öst, 2004), whereas others failed to identify such relationship (Nandi et al., 2004; Vinokur et al., 2011). These mixed findings suggest the need to examine this association more in depth.

**Hypothesis (4)** High psycho-physical symptoms and workload levels are negatively associated with job satisfaction among affected and non-affected workers over and above demographic variables. Perceived job satisfaction is negatively associated with PTSD symptoms and positively associated with coping self-efficacy among victimized workers, over and above psycho-physical symptoms and workload, and time since event.

Since victims with low CSE levels and high PTSD symptoms are less likely to recover spontaneously and more likely to experience a weaker decline in symptoms (Morina et al., 2014), they are more likely to require treatment. However, only one study to date has specifically investigated the relationship between CSE and help-seeking behavior (Bosmans and van der Velden, 2015). Past studies suggest that the severity of post-event mental health problems, particularly PTSD symptomatology, is the factor most consistently related to the utilization of mental health services (MHS) following trauma exposure (Boscarino et al., 2004; Gavrilovic et al., 2005; Elhai et al., 2006; van der Velden et al., 2007). Indeed, PTSD severity is related to increased use of MHS among residents of disaster affected areas (Frahm et al., 2013), survivors of terrorist attacks (Boscarino et al., 2004) and, more generally, victims of disasters (Van der Velden et al., 2006). Although trauma-exposed individuals are more likely to use MHS compared to non-affected people (Van der Velden et al., 1992, 2006), many victims do not utilize MHS or wait to seek treatment, even when they experience severe mental health problems (Kessler et al., 1998; Koenen et al., 2017). This may be due to different perceived barriers, such as stigma associated with mental health care (Gorman et al., 2011), low mental health literacy and helplessness related to ongoing symptoms (Davis et al., 2008; Ghafoori et al., 2014). With respect to robberies, a study among Dutch victims of bank robberies found that significantly more victims (32%) compared to non-victimized colleagues (9%) had used MHS, while the number of therapeutic sessions did not significantly differ between victims and non-victims who sought treatment (Van der Velden et al., 1992). To our knowledge, there are no studies available that have evaluated post-robbery and post-theft MHS utilization among victimized employees working in small, independently owned businesses.

Furthermore, because workload is positively associated with emotional exhaustion (Aronsson et al., 2017) which, in turn, may impair workers' mental health, those who perceive higher workload could be more likely to seek treatment than those who experience low workload. Indeed, previous research has found that poor mental health is an important factor predicting primary care consultation (Bellón et al., 2007). Edmond et al. (2013) found that the family doctor was the most frequently utilized service by the survivors of intimate partner violence. In Italy, the popularity of general practitioners (GP) can be explained by the fact that all citizens have access to this service free of charge. Furthermore, GPs play a gatekeeping role because they are the first point of contact for referrals to consultants, and are responsible for prescribing medication.

Since victims with low CSE and high PTSD symptoms are more likely to experience mental health complaints, we expected that they will be more likely to use MHS and consult their family doctor.

**Hypothesis (5)** The use of medical health services (MHS) by workers affected by thefts and/or robberies will be positively associated with post-traumatic symptoms and negatively associated with coping self-efficacy, over and above psycho-somatic symptoms and workload.

## Materials and methods

### Sample and procedure

This cross-sectional study was carried out with 492 workers employed in jewelers (*N* = 250) and tobacco shops (*N* = 242) throughout Italy in collaboration with their national trade unions. Respondents completed an anonymous self-report questionnaire. Tobacconists completed a paper-and-pencil questionnaire—since they are less likely to use the internet—whereas jewelers completed an online questionnaire.

As revealed by Table 1, the sample was equally distributed between jewelers and tobacconists who were the 50.81% and the 49.19% of the total sample respectively. The two professional categories were broadly similar as regards the main socio-demographic variables; with regard to the exposure to violent events, 31.90% of jewelers and 36.60% of tobacconists were victims of thefts, whereas 68.10% of jewelers and 63.40% of tobacconists were exposed to both thefts and robberies. Considering the similarities between the two professional categories, we considered them comparable, and we carried out all analyses on the combined sample. Considering the total sample, the majority of respondents were male (82.40%), married (76.40%), working with other co-workers (71.70%) and were owners of the shop (88.20%). The average age was 50.28 years (*SD* = 10.12) with an average job tenure of 29.04 years (*SD* = 11.29) and average tenure in their current position of 21.79 years (*SD* = 12.29). In the majority of cases, the shop was located in the immediate proximity of houses and other shops (94.20%) and open only during the day (67.20%). 182 shops were located in the North, 92 small business in the Centre and 200 in the South of Italy.

**Table 1:** Socio-demographic data compared for professional categories.

**Socio-demographic variables**	**Jewelers (*N* = 250)**	**Tobacconists (*N* = 242)**
	***n*** **(%)**	***n*** **(%)**
**GENDER**		
Male	202 (80.80%)	202 (84.20%)
Female	48 (19.20%)	38 (15.80%)
**MARITAL STATUS**		
Single	32 (12.80%)	39 (16.50%)
Married	195 (78.00%)	177 (74.70%)
Divorced	18 (7.20%)	16 (6.80%)
Widower	5 (2.00%)	5 (2.10%)
**WORK WITH**		
Always alone	25 (10.00%)	12 (5.00%)
Mainly alone	55 (22.00%)	47 (19.50%)
With one or more colleagues	170 (68.00%)	182 (75.50%)
**SHOP LOCATION**		
Isolated	5 (2.00%)	23 (9.60%)
Proximity of houses and shops	240 (98.00%)	217 (90.40%)
**SHOP LOCATION**		
Northern of Italy	90 (38.30%)	92 (38.50%)
Centre of Italy	40 (17.00%)	52 (21.80%)
Southern of Italy and Islands	105 (44.70%)	95 (39.70%)
**I WORK AS**		
Owner	222 (94.50%)	196 (82.00%)
Employee	13 (5.50%)	43 (18.00%)
	***M (SD)***	***M (SD)***
Age	51.60 (9.94)	48.96 (10.15)
Job tenure	31.00 (11.49)	27.05 (10.75)
Job tenure in the current position	26.00 (12.03)	17.53 (11.03)

In total, 319 workers had been exposed to robberies and/or thefts. More specifically, 108 workers were victims of thefts only, 211 were exposed to both thefts and robberies, and 173 workers didn't experience any events. Most respondents had been exposed to thefts in the past 13–24 months (80.30%), while more than half (56.70%) were victims of robberies in the past 7–12 months.

### Measurement

Participants who were exposed to thefts and/or robberies were invited to complete the whole questionnaire, whereas those who had not been exposed completed some sections only. Victims completed additional measures related to post-traumatic stress symptomatology, trauma related copy self-efficacy and use of mental health services.

Participants were asked whether they experienced thefts or robberies in their workplace. The two items were combined to a dichotomous measure of **exposure severity** to distinguish between respondents who were exposed to thefts only and those who reported multiple exposures (both thefts and robberies). Being exposed to both types of events was considered as more severe than being victims of a single type of event. In addition, participants were invited to indicate when the event took place (e.g., *How long ago was the last theft in your workplace?)* on a 5-point scale, ranging from 1 = *0–6 months ago* to 5 = *more than 24 months ago*.

#### General psychological health

General psychological health was assessed using the 12-item General Health Questionnaire (Goldberg, 1978, 1992; Goldberg and Williams, 1988; GHQ-12). This well-established screening instrument has been widely used in trauma research in different settings (e.g., Connor et al., 2006). It exists in several versions, but we decided to use the twelve-item version because of its good statistical properties (Goldberg and Williams, 1988). The questionnaire comprises three subscales: social dysfunction is assessed with six items related to difficulties in social performing and dealing with problems (e.g., “*Have you recently felt you couldn't overcome your difficulties?*”); general dysphoria evaluates the presence of psycho-somatic symptoms (four items, e.g., “*Have you recently been confidence in yourself?*”), and finally, loss of confidence assesses self-esteem levels (two items, e.g., “*Have you recently been losing confidence in yourself?*”). Responses are based on a four-point Likert scale that assesses if and how the individual's mental state differs from his or her usual state (from 0 = *better than usual/more so than usual* to 3 = *much less than usual* for positively worded items and for negatively 0 = *not at all* to 3 = *much more than usual* for negative worded items). For the purpose of this study, the total GHQ score was chosen over the three-dimensional model (with items loading exclusively on each GHQ factor), as the GHQ-12 was initially designed as a one-factor measure (Goldberg and Williams, 1988), and many researchers support the unidimensional use of the questionnaire (e.g., Shevlin and Adamson, 2005). This scale gives a total score ranging from 0 to 36 points, in which a greater score means a high level of malaise. In the present study, Cronbach's alpha for this scale was good (α = 0.80).

#### Job satisfaction

Job satisfaction was assessed using a single item measuring overall satisfaction (Giorgi et al., 2015b; e.g., *How satisfied have you been with your work?*). The responses were obtained on a ten-point scale (from 0 = *no satisfaction* to 10 = *satisfaction*) where a higher score indicates greater job satisfaction. This single item was used in previous studies focused on robberies (Giorgi et al., 2015b), showed sufficient validity and was positively related to more general scales of theoretically associated constructs (Faragher et al., 2005; Lapierre et al., 2005).

#### Social support seeking

Social support seeking as a coping strategy was assessed using Coping Orientation to Problems Experienced scale (COPE-IV; Sica et al., 2008). This instrument was used to assess participants' tendency to seek social support in the process of coping with problems (10 items; e.g., *I've been getting emotional support from others*). Cronbach's alpha for this scale was good (α = 0.88).

#### Workload

Workload perceptions were evaluated using a subscale taken from the Areas of Work life Survey (Leiter and Maslach, 2006) to capture the extent to which work demands spill into personal life as well as the physical and intellectual burden of job demands (e.g., *I do not have time to do the work that must be done*). The six items were assessed on a five-point Likert-type scale (from 1 = *strongly disagree* to 5 = *strongly agree*). As indicated by Maslach and Leiter (2008), scores greater than 3 denote a satisfactory alignment between the individual and the workplace's demands, whereas low scores reveal the presence of risk factors for the development of work-related stress. Cronbach's alpha for this scale was good (α = 0.81).

#### Post-traumatic stress symptoms

The six-item Impact of Event-Revised scale (IES-R; Thoresen et al., 2010) was used to assess symptoms of post-traumatic stress in response to a traumatic event. Participants were invited to indicate how frequently symptoms of intrusion (two items; e.g., *Other things kept making me think about it*), avoidance (two items; e.g., *I tried not to think about it*) and hyperarousal (two items; e.g., *I felt watchful or on-guard*; Weiss and Marmar, 1997) were experienced in the period following the last robbery or theft on a four-point scale (from 1 = *never* to 4 = *often*). Instead of using the three-dimensional solution, a global score (from 0 to 24) was calculated by adding the scores for each of the three subscales. This unidimensional solution gives an overall post-traumatic stress measure and it was found to be positively correlated with general health (Giorgi et al., 2015a). Cronbach's alpha for the IES scale was good (α = 0.88). Respondents were asked to take the (most recent) theft/robbery in mind when answering the questions on PTSD symptoms. In case respondents experienced both thefts and robberies, they were asked to focus on one of these events of their own choosing.

#### Trauma-related coping self-efficacy

Trauma-related coping self-efficacy (CSE) was assessed with a seven-item Coping self-efficacy scale (CSE-7; Bosmans et al., 2015b). Respondents rated their perceived efficacy in coping with diverse consequences derived from the theft or robbery (e.g., “*dealing with frightening images or events about the event*,” “*being emotionally strong*”) on a seven-point Likert scales (from 1 = *I am completely incapable of* to 7 = *I am perfectly capable of*). Cronbach's alpha for the CSE scale was good (α = 0.89). Respondents were asked to take the same event in mind as the event when they answered the questions about PTSD symptoms.

#### Use of (medical) health services

Use of (medical) health services was assessed using a single item *(“Have you contacted your general practitioner at any time during the past 12 months in connection with the last armed robbery/theft?”)*. Participants were invited to indicate on a ten-point Likert scale (from 1 = never to 10 = 9 or more times) how frequently, during the past 12 months, they have contacted their general practitioner to deal with consequences due to the last robbery or theft. Using the same scale, respondents answered whether, during the past 12 months, they have contacted a mental health professional (through public or private mental health services) to manage the consequences derived from theft and/or robbery exposure (e.g., *Have you been in contact with an independent psychiatrist, psychologist and/or psychotherapist for yourself during the past 12 months?*).

### Statistical analyses

The data were analyzed using SPSS, version 20. One-way analysis of variance (ANOVA) was carried out to verify the presence of differences in levels of psycho-somatic complaints, workload and social support, comparing victims of thefts and robberies with non-affected workers. Significant differences were subjected to multiple comparisons using Bonferroni's highly significant difference to understand the nature of the differences.

One-way analysis of covariance (ANCOVA) was used in order to analyze differences in levels of PTSD symptoms and CSE, comparing workers affected by thefts and robberies with victims of thefts only. ANCOVA was also used to explore differences in the number of GP visits in the past 12 months, comparing non-affected individuals with those who were affected by thefts only and those who were exposed to both thefts and robberies.

Multivariate regression analyses were carried out to verify the influence exerted by psycho-physical symptoms and workload on job satisfaction among affected and non-affected workers. Furthermore, regressions were used to assess the influence of psycho-physical and post-traumatic symptoms, coping self-efficacy and workload on job satisfaction among affected workers.

A *p* value < 0.05 was considered as statistically significant.

## Results

The means, standard deviations, intercorrelations, skewness and kurtosis statistics, and internal consistencies (Cronbach's alpha) for the measures used in this study are provided in Tables 2, 3 (for non-affected and affected subjects, respectively).

**Table 2:** Descriptive, internal consistency, and intercorrelations for study variables among non-affected workers (*N* = 173).

**Measure**	***M***	***SD***	**Skewness**	**Kurtosis**	**1**	**2**	**3**
1. Psycho-physical health	2.92	1.48	0.65	−0.30	**0.80**		
2. Social support seeking	2.08	0.61	0.55	0.34	0.32^**^	**0.87**	
3. Workload	3.19	0.80	−0.05	−0.58	0.12	−0.17^*^	**0.79**

Boldfaced numbers on the diagonal represent Cronbach's alpha;

* *p < 0.05*,

** *p < 0.01*.

**Table 3:** Descriptive, internal consistency, and intercorrelations for study variables among affected workers (*N* = 319).

**Measure**	***M***	***SD***	**Skewness**	**Kurtosis**	**1**	**2**	**3**	**4**	**5**
1. Psycho-physical health	**3.37**	**1.55**	0.66	0.42	**0.80**				
2. Social support seeking	2.20	0.65	0.61	0.07	0.40^**^	**0.88**			
3. Workload	3.58	0.80	−0.35	0.03	0.28^**^	0.10	**0.80**		
4. CSE	4.85	1.36	−0.39	−0.14	−0.08	0.06	0.06	**0.89**	
5. PTSD symptoms	2.18	1.01	−0.26	−0.73	0.26^**^	0.29^**^	0.20^**^	−0.27^**^	**0.88**

Boldfaced numbers on the diagonal represent Cronbach's alpha;

** *p < 0.01*.

With regard to Hypothesis 1, as expected *post-hoc* comparisons showed that victims of both thefts and robberies experienced higher levels of psycho-somatic symptoms [*F*_(2, 486)_ = 6.65; *p* < 0.01], workload [*F*_(2, 485)_ = 13.49; *p* < 0.001] and seeking social support [*F*_(2, 486)_ = 4.47; *p* < 0.05], compared to non-affected colleagues (all Cohen's D between 0.18 and 0.51) (see Table 4).

**Table 4:** ANOVA results for workload and social support seeking among the three study groups.

**Variables**	**Groups**	**Mean**	***SD***	***F*^a^**
Psycho-somatic symptoms	No event	2.92	1.48	6.65^**^
	Thefts	3.15	1.42	
	Thefts and robberies	3.49	1.61	
Workload	No event	3.19	0.80	13.49^***^
	Thefts	3.53	0.72	
	Thefts and robberies	3.61	0.84	
Social support seeking	No event	2.08	0.61	4.47^**^
	Thefts	2.08	0.66	
	Thefts and robberies	2.25	0.64	
Job satisfaction	No event	6.88	1.99	12.14^***^
	Thefts	6.46	2.12	
	Thefts and robberies	5.74	2.23	

** *p < 0.01*,

*** *p < 0.001*.

a *DF = 2*.

With regard to hypotheses 2 and 3, one-way analysis of covariance (ANCOVA) showed that workers affected by thefts and robberies had significantly higher PTSD-symptom levels [*F*_(1, 303)_ = 22.43; *p* < 0.001; Cohen's *D* = −0.58] and significant lower coping self-efficacy levels, compared to workers affected by thefts only [*F*_(1, 296)_ = 8.37; *p* < 0.01; Cohen's *D* = 0.36; see Table 5).

**Table 5:** ANCOVA results for PTSD symptoms and coping sel-efficacy among affected groups.

**Variables**	**Groups**	**Mean**	***SD***	***F*^*Time*^^a^**	***F*^*groups*^^a^**
**PTSD symptoms**	Thefts	5.36	2.94	1.75	22.43^***^
	Thefts and robberies	5.19	1.34		
**Coping self-efficacy**	Thefts	5.14	2.93	5.50^*^	8.37^***^
	Thefts and robberies	4.71	1.34		

a *DF = 1*.

* *p < 0.05*,

*** *p < 0.001*.

*Covariate: time since event*.

According to hypothesis 4, we expected that the presence of psycho-physical symptoms and the perception of high workload would be negatively associated with job satisfaction (while controlling for gender, age and marital status). The results of the multivariate regression analyses with workload and psycho-somatic symptoms as predictors of job satisfaction were statistically significant for non-affected workers only (see Table 6). The full model explained 39% of total variance, but when controlling for gender, age and marital status, only psycho-physical symptoms level was significantly and strongly related to job satisfaction **(**Δ*R*^2^ = 0.29; β = −0.56; *p* < 0.001; Cohen's *f* ^2^ = 0.64). The perception of high workload was not associated with the job satisfaction levels of workers who had not been affected by assessed traumatic events.

**Table 6:** Summary of regression analyses for variables predicting job satisfaction in non-affected group.

				**Non-affected (N**^**max**^ = **163)**		
**Variable**		**Δ*R^2^***	***f^2^***	**β**	***t***	***P***
Step 1		0.10				
	Gender			−0.32	−4.10	0.00
	Age			−0.01	−0.10	0.92
	Marital status			−0.01	−0.09	0.93
Step 2		0.29	0.64			
	Gender			−0.20	−3.06	0.00
	Age			0.05	0.69	0.49
	Marital status			−0.02	−0.31	0.76
	Workload			0.09	1.37	0.17
	GHQ total score			−0.56	−8.39	0.00

In addition, according to hypothesis 4, we expected that job satisfaction would be negatively related to post-traumatic symptoms and positively related to coping self-efficacy in workers affected by thefts and/or robberies, over and above the effects of psycho-somatic symptoms and workload (controlling for gender, age, marital status and time since event). The results show that the factor highly related to job satisfaction in workers affected by thefts and/or robberies was psycho-somatic symptoms level (β = −0.31; *p* < 0.001); the level of coping self-efficacy (β = 0.20; *p* < 0.01) was significantly related to job satisfaction, explaining a limited amount of variance while controlling for psycho-somatic symptoms (see Table 7). Post-traumatic symptoms were not independently associated with job satisfaction.

**Table 7:** Summary of regression analyses for variables predicting job satisfaction in affected group.

				**Affected (N**^**max**^ = **303)**		
**Variable**		**ΔR^2^**	***f^2^***	**β**	***t***	***p***
Step 1		0.01				
	Gender			0.02	0.31	0.76
	Age			−0.08	−1.28	0.20
	Marital status			0.10	1.64	0.10
Step 2		0.11	0.14			
	Gender			0.05	0.86	0.39
	Age			−0.06	−1.08	0.28
	Marital status			0.10	1.73	0.08
	Workload			−0.04	−0.68	0.50
	GHQ total score			−0.32	−5.57	0.00
Step 3		0.03	0.18			
	Gender			0.04	0.67	0.49
	Age			−0.01	−0.22	0.82
	Marital status			0.09	1.54	0.12
	Workload			−0.05	−0.95	0.34
	GHQ total score			−0.31	−5.28	0.00
	PTSD symptoms			0.02	0.28	0.78
	Coping self-efficacy			0.20	3.27	0.00
	Time since event			−0.01	−0.15	0.88

Finally, regarding hypothesis 5, the results of ANCOVA showed no significant differences in the number of GP visits in the past 12 between non-affected individuals (*M* = 1.88), those affected by thefts only (*M* = 2.24) and those exposed to both thefts and robberies (*M* = 2.23). Differences in the number of event-related GP-visits between thefts (*M* = 1.02) and thefts and robberies (*M* = 1.30) did not reach the *p* < 0.05 significance level between (*p* = 0.06) while controlling for gender, age and marital status.

With respect to the use of mental health services in the past 12 months, multivariate logistic regression analyses showed no significant differences between non-affected (9.8%), those affected by thefts (7.4%) and those affected by thefts and robberies (9%).

## Discussion

This cross-sectional study focuses on psychological consequences of robberies and thefts, an increasing health and safety issue in Italy, among the under-investigated population of jewelers and tobacconists. Although some studies have investigated the consequences of being exposed to robberies and thefts on workers' well-being, post-traumatic stress, psycho-physical symptoms and job satisfaction (e.g., Giorgi et al., 2015b; Mucci et al., 2015), the ways in which experiencing these PTEs at work may affect workers' perceptions remains unclear. This study assessed the associations between exposure to robberies and thefts with psychological well-being in light of the solid scientific framework of the Conservation of Resource Theory (Hobfoll, 1989; Halbesleben et al., 2014).

Firstly, this study confirmed the hypotheses 1, 2, and 3. Victims of multiple violence (both thefts and robberies) reported higher levels of psycho-somatic complaints, greater workload perceptions and higher levels of social support seeking compared to non-affected counterparts. Additionally, they experienced higher levels of PTSD symptoms and weaker CSE perceptions compared to those who were exposed to thefts only. According to the COR model, the higher the threat of loss or actual loss of resources, the more harmful are the negative psycho-social consequences. Workers who are victims of both thefts and robberies seem to be more likely to perceive a threat to their working conditions, feeling less safe within their workplace and needing more resources to face the trauma and its aftermath, than their counterparts. Consequently, they tend to experience more serious resource depletion, which may result in a reduced capacity to manage everyday work demands, the need for compensatory resource replacement and the risk of being stressed by the negative balance between energies invested in dealing with recovery demands and poor benefits achieved. As a result, they are more likely to perceive higher workload, search for greater social support and experience a more serious psycho-physical impairment than their non-exposed counterpart. Furthermore, workers may experience trauma re-actualization when they are victims of both thefts and robberies, increasing post-traumatic symptoms (Van der Kolk, 1989; Schwartz et al., 2005; Wisnivesky et al., 2011; Yuan et al., 2013). Indeed, the reoccurrences of violence may stimulate hopelessness, which may influence workers' health (Herschcovis and Barling, 2010). Moreover, literature found associations between perceived helplessness and post-traumatic complaints, since victims of violence seem to commonly feel helpless or fearful even after a robbery (Mucci et al., 2015). Consequently, it is possible that employees who were exposed to robberies are more likely to experience feelings of helpless or fearful and, in turn, they may be at higher risk of developing PTSD symptoms.

In addition, the development of fears (for instance of being a victim of future robberies) might decrease CSE levels. For example, Di Giacinto et al. (2014) pointed out that fear is an important factor that influences the extent to which robbery victims experience post-traumatic symptoms. Van der Velden et al. (1992) showed that victims of bank robberies are more afraid of future robberies than non-victims.

Accordingly, workers affected by violence reported impediments in self-management, a factor related to self-efficacy (Giorgi et al., 2016). Furthermore, CSE might be impaired by the level of stress perceived during or immediately after the trauma (Bosmans and van der Velden, 2015; Bosmans et al., 2015c). In line with COR theory, workers who are exposed to both thefts and robberies, which are perceived as fearful and stressful events, continue to be affected by resource loss and, thus, they are more likely to feel like they don't have the necessary energies and capacities to successfully manage the negative consequences following traumas, experiencing higher PTSD symptoms and lower CSE levels.

Hypothesis 4 was not confirmed. In non-affected workers, job satisfaction was associated to workload and psycho-somatic symptoms. However, when controlling for socio-demographic variables, only psycho-somatic symptoms were related to job satisfaction in this cohort. Among those who were exposed to both thefts and robberies, PTSD symptomatology and high workload were not independently related to job satisfaction, whereas CSE levels explained a small amount of variance in satisfaction, over and above all other predictors. The differences regarding workload found between the two groups could be explained by the fact that those affected by violence might have learned, to a certain extent, to cope with the trauma by becoming more resilient. In line with this reasoning, post-traumatic growth - which may stem from the struggle with the trauma - has been found to be associated with the development of coping skills and the redefinition of life in a more meaningful way (Williams et al., 2003; Baillie et al., 2014).

Similar to findings regarding non-exposed workers, psycho-somatic symptoms represent the variables more strongly related to job satisfaction in workers exposed to thefts or robberies (or both). Indeed, job satisfaction is affected by psycho-somatic complaints, as shown by previous research (Faragher et al., 2005; Alexopoulos et al., 2014). The fact that the presence of post-traumatic symptoms did not influence job satisfaction is in line with some previous findings (Nandi et al., 2004; Vinokur et al., 2011). This might be due to the fact that in our sample, some time had elapsed since the trauma for the majority of the sample (as confirmed by reported time since the last theft or robbery) and, as a result, its effect might be attenuated by natural course of coping. Robbery puts demands on workers - often company owners - to remain resilient and cope in a positive way.

In conclusion, hypothesis 5 was not confirmed: no differences were found in (mental) health services utilization. There might be several explanations for this finding. Possibly cultural aspects might emerge: discrimination and stigma potentially associated with a mental health diagnosis or receiving mental health treatment may be responsible for delays in seeking treatment (Gorman et al., 2011). Thus, in Italy—like in other countries—some workers might be still reluctant to use mental health services (Kessler et al., 1998; Koenen et al., 2017). This may explain the fact that affected and non-affected workers did differ in mental health problems, while no differences were found in MHS utilization. Since treatment seeking victims tend to have lower CSE perceptions than nontreatment seeking individuals (Bosmans et al., 2016), an alternative explanation could be that victims didn't seek treatment because they had high overall CSE levels. It would be interesting to test this hypothesis in future. From an applied perspective, even if no significant differences were found in mental health problems and services utilization between affected and non-affected workers, it is noteworthy that therapists may help victims to reduce negative effects deriving from the exposure to PTEs at work. Indeed, several psychological interventions aimed at individuals who experienced a trauma have been designed to mitigate acute distress reactions with the goal of preventing the development of chronic PTSD symptoms (Foa et al., 2005). Such interventions include both traditional individual crisis interventions (e.g., prompt intervention aimed at comforting victims, mobilizing their resources to react to the traumatic experience; Setti and Argentero, 2016) and group psychological debriefing, especially Critical Incident Stress Debriefing (i.e., small-group discussion implemented to assist individuals in reaching a sense of closure post crisis and encourage them to discuss and ventilate intense emotions facilitating their processing of their responses to the trauma; Mitchell and Everly, 2000). Furthermore, brief (i.e., 4–5 sessions) cognitive-behavioral therapy (Bryant et al., 1998) and medication (e.g., benzodiazepines, propranolol, and hydrocortisone; Pitman et al., 2002) may be useful to support victims who exhibit malaise symptoms.

## Strengths and limitations

The current study has a number of strengths. It gives an original contribution to the existing literature of psychological sequelae following robbery and theft exposure: to date, this is one of the first studies with a large sample of workers employed in small businesses assessing how experiencing these PTEs may impact on their well-being and job satisfaction. In addition, to the best of our knowledge, this is the first study to compare the differences in terms of PTSD symptoms, by coping self-efficacy perceptions, social support and treatment seeking, between workers who were exposed to both thefts and robberies and those who were victims of thefts only.

A further strength is the focus on an understudied and difficult-to-track population, since only a few studies have concentrated on workers employed in small businesses while previous research has predominantly focused on other professional categories, such as bank tellers (e.g., Armour and Hansen, 2015; Mucci et al., 2015).

However, the findings are also subjected to several limitations. First, since the majority of our subjects were men, and gender has been found to affect the levels of mental health and PTSD symptoms (see Christiansen and Hansen, 2015), this might have partially influenced our findings. However, the gender distribution in our sample is representative of these worker cohorts in the Italian context. Secondly, causal relationships cannot be inferred since the study design was cross-sectional. Consequently, further research should adopt longitudinal design and assess workers' well-being and job satisfaction before violent episodes take place, in order to more thoroughly interpret how experiencing robberies and thefts may impact on these psychological states. Using a longitudinal design, it will be beneficial to examine the potential moderation role exerted by resources (for example, in terms of CSE) in the relationship between the exposure to PTEs (i.e., thefts and robberies), and individual and occupational outcomes (respectively in terms, for example, of health symptoms and job satisfaction), among exposed subjects.

Furthermore, this study used only self-report measures for data gathering which might contribute to common method bias. Therefore, a further limitation was that the assessment of post-traumatic stress symptomatology was based on self-reporting without a clinical examination. Although IES score has a good accuracy showing a high correlation with PTSD diagnosis, this scale does not include all criteria for PTSD as stated by DSM-5. Further research should collect information from other sources rather than rely solely on self-reports: clinical interviews should be integrated to assess PTSD symptomatology.

Furthermore, data on pre- and peri-trauma variables were not gathered, although it has been found that they may affect individuals' susceptibility to PTSD. Thus, it has been shown that both pre-traumatic risk factors—such as the presence of pre-event mental health problems; (Van der Velden et al., 2016); co-existing psychopathological disorders; (Skogstad et al., 2013); or having experienced prior non-occupational traumatic life events; (Hansen and Elklit, 2011)—and peri-traumatic factors—such as perceived threat to one's life, helplessness and the presence of a weapon; (Giorgi et al., 2015a)—might increase the likelihood of developing PTSD. Therefore, controlling for these confounding variables is suggested going forward.

The fact that results were based on a sample of jewelers and tobacconists means that they may not be generalizable to other working populations. Replications should be conducted in other professional categories. Moreover, an important endeavor would be to examine whether these findings replicate in other cultural contexts through comparative studies.

Another limitation is due to the fact that we received a response from a representative sample from each category of theft and robbery related violence, although possible selection bias due to the voluntary participation into the research cannot be ruled out. It is possible that those who experienced robbery and theft-related violence were more motivated to respond and, as such, are overrepresented. Alternatively, non-response to a survey of this type may be due to avoidance symptoms associated with post-traumatic stress.

Finally, although job satisfaction was measured by a well-validated single item, the use of facet measures would make it possible to identify specific areas that might be differently influenced by trauma exposure. Future studies should attempt to study different aspects of job satisfaction and examine other behavioral work-related outcomes of robbery and theft exposure (e.g., work performance). In addition, future research should also focus on the influence of possible compensations from insurance companies, or problems with receiving compensations, on post-event mental health problems, workload and job satisfaction (O'Donnell et al., 2015).

## Conclusion

In conclusion, thefts and/or robberies exposure undermines differently workers' well-being and job satisfaction. Workers who were exposed to both types of events were more likely to develop PTSD symptomatology and impaired perceived ability to cope with the trauma, when compared to those who were exposed to thefts only. Victims of multiple violence reported greater workload, higher psycho-somatic complaints and greater tendency to seek social support in comparison with their unexposed colleagues. PTSD symptomatology was not significantly associated with job satisfaction. By contrast, CSE perceptions explained a limited amount of variance of satisfaction, although psycho-somatic impairment was the main predictor of job satisfaction both in affected and non-affected workers. Investigating these effects is crucial to formulate preventive measures and tailored interventions for victims.

## Ethics statement

We have investigated topics related to occupational well-being and work environment/conditions without any reference to private/personal issues. All participants were employees of private businesses, and they can be classified as “normal subjects” (i.e., without specific mental health problems): they were all adult mentally fit. Italian law does not impose these requirements (i.e., ethics approval and written informed consent) in case of these type of self-report and anonymous research carried out on healthy subjects.

## Author contributions

IS gave substantial contribution to the design of the work (acquisition and interpretation of the data); in drafting the work and revising it critically; gave the final approval of the version to be published; agrees to be accountable. PvdV gave substantial contribution to the analysis and interpretation of the data; in drafting the work; in revising the work critically; gave the final approval of the version to be published; agrees to be accountable. VS gave substantial contribution to the design of the work (acquisition and interpretation of the data); in revising the work critically; gave the final approval of the version to be published; agrees to be accountable. MF gave contribution to the data analysis; gave the final approval of the version to be published; agrees to be accountable. GG gave contribution to the design of the work (acquisition and interpretation); gave the final approval of the version to be published; agrees to be accountable. DO gave contribution to the data analysis; in revising the work critically; gave the final approval of the version to be published; agrees to be accountable. PA gave substantial contribution to the design of the work (acquisition and interpretation); in revising the work critically; gave the final approval of the version to be published; agrees to be accountable.

### Conflict of interest statement

The authors declare that the research was conducted in the absence of any commercial or financial relationships that could be construed as a potential conflict of interest.

## References

[B1] Aebi M. F. Aromaa K. de Cavarlay B. A. Barclay G. Gruszczynska B. von Hofer H. (2010). European Sourcebook of Crime and Criminal Justice Statistics. Den Haag, NL: Boom Juridische Uitgevers.

[B2] Alexopoulos E. C. Palatsidi V. Tigani X. Darviri C. (2014). Exploring stress levels, job satisfaction, and quality of life in a sample of police officers in Greece. Saf. Health Work 5, 210–215. 10.1016/j.shaw.2014.07.004 25516814 PMC4266800

[B3] American psychiatric association I. (2013). Diagnostic and Statistical Manual of Mental Disorders (DSM-5®). Washington, DC: American Psychiatric Pub.

[B4] Armour C. Hansen M. (2015). Assessing DSM-5 latent subtypes of acute stress disorder dissociative or intrusive? Psychiatry Res. 225, 476–483. 10.1016/j.psychres.2014.11.063 25535010

[B5] Aronsson G. Theorell T. Grape T. Hammarström A. Hogstedt C. Marteinsdottir I.. (2017). A systematic review including meta-analysis of work environment and burnout symptoms. BMC Public Health 17:264. 10.1186/s12889-017-4153-7 28302088 PMC5356239

[B6] Baillie S. E. Sellwood W. Wisely J. A. (2014). Post-traumatic growth in adults following a burn. Burns 40, 1089–1096. 10.1016/j.burns.2014.04.007 25037992

[B7] Bandura A. (1997). Self-Efficacy: The Exercise of Control. New York, NY: W. H. Freeman.

[B8] Belleville G. Marchand A. St-Hilaire M. H. Martin M. Silva C. (2012). PTSD and depression following armed robbery: patterns of appearance and impact on absenteeism and use of health care services. J. Trauma Stress 25, 465–468. 10.1002/jts.21726 22833477

[B9] Bellón J. A. Delgado-Sánchez A. de Dios Luna J. Lardelli-Claret P. (2007). Patient psycho-social factors and primary care consultation: a cohort study. Fam. Pract. 24, 562–569. 10.1093/fampra/cmm059 17906310

[B10] Benight C. C. Bandura A. (2004). Social cognitive theory of post-traumatic recovery: the role of perceived self-efficacy. Behav. Res. Ther. 42, 1129–1148. 10.1016/j.brat.2003.08.008 15350854

[B11] Benight C. C. Flores J. Tashiro T. (2001). Bereavement coping self-efficacy in cancer widows. Death Stud. 25, 97–125. 10.1080/074811801461900 11708355

[B12] Benight C. C. Ironson G. Klebe K. Carver C. S. Wynings C. Burnett K. (1999). Conservation of resources and coping self-efficacy predicting distress following a natural disaster: a causal model analysis where the environment meets the mind. Anxiety Stress Coping 12, 107–126. 10.1080/10615809908248325

[B13] Benka J. Nagyova I. Rosenberger J. Macejova Z. Lazurova I. Van der Klink J. (2014). Is coping self-efficacy related to psychological distress in early and established rheumatoid arthritis patients?. J. Dev. Phys. Disabil. 26, 285–297. 10.1007/s10882-013-9364-y

[B14] Boscarino J. A. Galea S. Adams R. E. Ahern J. Resnick H. Vlahov D. (2004). Mental health service and medication use in New York City after the September 11, 2001, terrorist attack. Psychiatr. Serv. 55, 274–283. 10.1176/appi.ps.55.3.274 15001728

[B15] Bosmans M. W. G. Komproe I. H. Van Loey N. E. Van der Knaap L. M. Benight C. C. van der Velden P. G. (2015b). Assessing perceived ability to cope with trauma: a multigroup validity study of a 7 item coping self-efficacy scale. Eur. J. Psychol. Assess. 33, 55–64. 10.1027/1015-5759/a000266

[B16] Bosmans M. W. van der Velden P. G. (2015). Longitudinal interplay between post-traumatic stress symptoms and coping self-efficacy: a four-wave prospective study. Soc. Sci. Med. 134, 23–29. 10.1016/j.socscimed.2015.04.007 25875423

[B17] Bosmans M. W. Hofland H. W. De Jong A. E. Van Loey N. E. (2015a). Coping with burns: the role of coping self-efficacy in the recovery from traumatic stress following burn injuries. Int. J. Behav. Med. 38, 642–651. 10.1007/s10865-015-9638-1 25851608 PMC4496529

[B18] Bosmans M. W. Van der Knaap L. M. Van der Velden P. G. (2015c). Personality traits as predictors of trauma-related coping self-efficacy: a three-wave prospective study. Pers. Individ. Dif. 76, 44–48. 10.1016/j.paid.2014.11.052

[B19] Bosmans M. W. Van der Knaap L. M. Van der Velden P. G. (2016). The predictive value of trauma-related coping self-efficacy for posttraumatic stress symptoms: differences between treatment-seeking and non–treatment-seeking victims. Psychol. Trauma 8, 241–248. 10.1037/tra0000088 26460493

[B20] Bowling N. A. Alarcon G. M. Bragg C. B. Hartman M. J. (2015). A meta-analytic examination of the potential correlates and consequences of workload. Work Stress 29, 95–113. 10.1080/02678373.2015.1033037

[B21] Bryant R. A. Harvey A. G. Dang S. T. Sackville T. Basten C. (1998). Treatment of acute stress disorder: a comparison of cognitive-behavioral therapy and supportive counseling. J. Consult. Clin. Psychol. 66, 862–866. 10.1037/0022-006X.66.5.862 9803707

[B22] Casteel C. Peek-Asa C. Greenland S. Chu L. D. Kraus J. F. (2008). A study of the effectiveness of a workplace violence intervention for small retail and service establishments. J. Occup. Environ. Med. 50, 1365–1370. 10.1097/JOM.0b013e3181845fcf 19092491

[B23] Christiansen D. M. Hansen M. (2015). Accounting for sex differences in PTSD: a multi-variable mediation model. Eur. J. Psychotraumatol. 6:26068. 10.3402/ejpt.v6.26068 25604705 PMC4300366

[B24] Connor K. M. Foa E. B. Davidson J. R. (2006). Practical assessment and evaluation of mental health problems following a mass disaster. J. Clin. Psychiatry 67, 26–33. 16602812

[B25] Davis R. G. Ressler K. J. Schwartz A. C. Stephens K. J. Bradley R. G. (2008). Treatment barriers for low-income, urban African Americans with undiagnosed post-traumatic stress disorder. J. Trauma Stress 21, 218–222. 10.1002/jts.20313 18404649 PMC2444044

[B26] Di Giacinto A. Brunetti M. Sepede G. Ferretti A. Merla A. (2014). Thermal signature of fear conditioning in mild post-traumatic stress disorder. Neuroscience 266, 216–223. 10.1016/j.neuroscience.2014.02.009 24561216

[B27] Edmond T. Bowland S. Yu M. (2013). Use of mental health services by survivors of intimate partner violence. Soc. Work Ment. Health 11, 34–54. 10.1080/15332985.2012.734180

[B28] Elhai J. D. Patrick S. L. Anderson S. Simons J. S. Frueh B. C. (2006). Gender-and trauma-related predictors of use of mental health treatment services among primary care patients. Psychiatry Serv. 57, 1505–1509. 10.1176/ps.2006.57.10.1505 17035574

[B29] European Institute for Crime Prevention Control International Statistics on Crime Justice (2011). Aggregates Compiled by NationMaster. Available online at: http://www.nationmaster.com/country-info/stats/Crime/Robberies

[B30] Faragher E. B. Cass M. Cooper C. L. (2005). The relationship between job satisfaction and health: a meta-analysis. Occup. Environ. Med. 62, 105–112. 10.1136/oem.2002.006734 15657192 PMC1740950

[B31] Foa E. B. Cahill S. P. Boscarino J. A. Hobfoll S. E. Lahad M. McNally R. J.. (2005). Social, psychological, and psychiatric interventions following terrorist attacks: recommendations for practice and research. Neuropsychopharmacology 30, 1806–1817. 10.1038/sj.npp.1300815 16012536

[B32] Frahm K. A. Barnett S. D. Brown L. M. Hickling E. J. Olney R. Campbell R. R. (2013). Post-traumatic stress disorder and use of psychiatric and alcohol related services: the effect of the 2004–2005 Florida hurricane seasons on veterans. Commun. Ment. Health J. 49, 636–642. 10.1007/s10597-012-9558-2 23054158

[B33] Gavrilovic J. J. Schützwohl M. Fazel M. Priebe S. (2005). Who seeks treatment after a traumatic event and who does not? A review of findings on mental health service utilization. J. Trauma Stress 18, 595–605. 10.1002/jts.20068 16382432

[B34] Ghafoori B. Barragan B. Palinkas L. (2014). Mental health service use after trauma exposure: a mixed methods study. J. Nerv. Ment. Dis. 202, 239–246. 10.1097/NMD.0000000000000108 24566510 PMC3959109

[B35] Giorgi G. Perez F. S. F. D'Antonio A. C. Mucci N. Ferrero C. Cupelli V.. (2015a). Psychometric properties of the impact of event scale-6 in a sample of victims of bank robbery. Psychol. Res. Behav. Manag. 8, 99–104. 10.2147/PRBM.S73901 25878514 PMC4388003

[B36] Giorgi G. Perez J. L. Montani F. Courcy F. Arcangeli G. (2015b). Distress and job satisfaction after robbery assaults: a longitudinal study. Occup. Med. 65, 290–295. 10.1093/occmed/kqv051 25972610

[B37] Giorgi G. Perminiene M. Montani F. Fiz-Perez J. Mucci N. Arcangeli G. (2016). Detrimental effects of workplace bullying: impediment of self-management competence via psychological distress. Front. Psychol. 7:60. 10.3389/fpsyg.2016.00060 26913013 PMC4753400

[B38] Goldberg D. (1978). Manual of the General Health Questionnaire. Windsor: NFER-Nelson.

[B39] Goldberg D. P. (1992). General Health Questionnaire (GHQ-12). Windsor: NFER-Nelson.

[B40] Goldberg D. P. Williams P. (1988). A User's Guide to the GHQ. Windsor: NFER-Nelson.

[B41] Gorman L. A. Blow A. J. Ames B. D. Reed P. L. (2011). National guard families after combat: mental health, use of mental health services, and perceived treatment barriers. Psychiatry Serv. 62, 28–34. 10.1176/ps.62.1.pss6201_0028 21209296

[B42] Halbesleben J. R. Neveu J. P. Paustian-Underdahl S. C. Westman M. (2014). Getting to the “COR” understanding the role of resources in conservation of resources theory. J. Manage. 40, 1334–1364. 10.1177/0149206314527130

[B43] Hansen M. Elklit A. (2011). Predictors of acute stress disorder in response to bank robbery. Eur. J. Psychotraumatol. 2:5864. 10.3402/ejpt.v2i0.5864 22893821 PMC3402148

[B44] Hansen M. Elklit A. (2013). Does acute stress disorder predict post-traumatic stress disorder following bank robbery? J. Interpers. Violence 28, 25–44. 10.1177/0886260512448848 22829214

[B45] Hansen M. Elklit A. (2014a). Who develops PTSD following bank robbery? A national cohort study, in Banking: Performance, Challenges and Prospects for Development, ed Simmons J. P. (New York, NY: Nova Publishers), 2562.

[B46] Hansen M. Elklit A. (2014b). Who develops post-traumatic stress symptoms following a bank robbery? in Banking: Performance, Challenges and Prospects for Development, ed Simmons J. P. (Hauppauge, NY: Nova Science Publishers Incorporated), 25–62.

[B47] Hansen M. Armour C. Elklit A. (2012). Assessing a dysphoric arousal model of acute stress disorder symptoms in a clinical sample of rape and bank robbery victims. Eur. J. Psychotraumatol. 3:18201. 10.3402/ejpt.v3i0.18201 22893845 PMC3402157

[B48] Hansen M. Armour C. Shevlin M. Elklit A. (2014). Investigating the psychological impact of bank robbery: a cohort study. J. Anxiety Disord. 28, 454–459. 10.1016/j.janxdis.2014.04.005 24846493

[B49] Herschcovis M. S. Barling J. (2010). Towards a multi-foci approach to workplace aggression: a meta-analytic review of outcomes from different perpetrators. J. Organ. Behav. 31, 24–44. 10.1002/job.621

[B50] Hobfoll S. E. (1989). Conservation of resources: a new attempt at conceptualizing stress. Am. Psychol. 44:513. 10.1037/0003-066X.44.3.513 2648906

[B51] Istat (2015). Delitti Denunciati Dalle Forze di Polizia all'autorità Giudiziaria. Available Online at: http://dati.istat.it/Index.aspx?DataSetCode=dccv_delittips

[B52] Judge T. A. Thoresen C. J. Bono J. E. Patton G. K. (2001). The job satisfaction–job performance relationship: a qualitative and quantitative review. Psychol. Bull. 127, 376–407. 10.1037/0033-2909.127.3.376 11393302

[B53] Kamphuis J. H. Emmelkamp P. M. (1998). Crime-related trauma: psychological distress in victims of bank robbery. J. Anxiety Disord. 12, 99–208. 10.1016/S0887-6185(98)00009-7 9653679

[B54] Karam E. G. Friedman M. J. Hill E. D. Kessler R. C. McLaughlin K. A. Petukhova M.. (2014). Cumulative traumas and risk thresholds: 12-month PTSD in the World Mental Health (WMH) surveys. Depress. Anxiety 31, 130–142. 10.1002/da.22169 23983056 PMC4085043

[B55] Kent G. (1987). Self-efficacious control over reported physiological, cognitive and behavioural symptoms of dental anxiety. Behav. Res. Ther. 25, 341–347. 10.1016/0005-7967(87)90012-X 3689292

[B56] Kent G. Gibbons R. (1987). Self-efficacy and the control of anxious cognitions. J. Behav. Ther. Exp. Psychiatry 18, 33–40. 10.1016/0005-7916(87)90069-3 3558850

[B57] Kessler R. C. Olfson M. Berglund P. A. (1998). Patterns and predictors of treatment contact after first onset of psychiatric disorders. Am. J. Psychiatry 155, 62–69. 10.1176/ajp.155.1.62 9433340

[B58] Koenen K. C. Ratanatharathorn A. Ng L. McLaughlin K. A. Bromet E. J. Stein D. J. Karam E. G.. (2017). Posttraumatic stress disorder in the World Mental Health Surveys. Psychol. Med. 47, 2260–2274. 10.1017/S0033291717000708 28385165 PMC6034513

[B59] Kraaij V. Garnefski N. Maes S. (2002). The joint effects of stress, coping, and coping resources on depressive symptoms in the elderly. Anxiety Stress Coping 15, 163–177. 10.1080/10615800290028468

[B60] Lambert J. E. Benight C. C. Harrison E. Cieslak R. (2012). The firefighter coping self-efficacy scale: measure development and validation. Anxiety Stress Coping 25, 79–91. 10.1080/10615806.2011.567328 21476153

[B61] Lapierre L. M. Spector P. E. Leck J. D. (2005). Sexual versus nonsexual workplace aggression and victims' overall job satisfaction: a meta-analysis. J. Occup. Health Psychol. 10, 155–169. 10.1037/1076-8998.10.2.155 15826225

[B62] Lattanzi G. (2010). Codice Penale. Rassegna di Giurisprudenza e di Dottrina. Milano, IT: Guffrè Editore.

[B63] Law F. M. Guo G. J. (2016). Correlation of hope and self-efficacy with job satisfaction, job stress, and organizational commitment for correctional officers in the taiwan prison system. Int. J. Offender Ther. Comp. Criminol. 60, 1257–1277. 10.1177/0306624X15574997 25805714

[B64] Leiter M. P. Maslach C. (2006). Areas of Worklife Survey Manual. Wolfville, NS: Center for Organizational Research and Development.

[B65] Lepore S. J. (2001). A social-cognitive processing model of emotional adjustment to cancer, in Psycho-Social Interventions for Cancer eds Baum A. Andersen B. L. (Washington, DC: American Psychological Association), 99–116.

[B66] Maslach C. Leiter M. P. (2008). Early predictors of job burnout and engagement. J. Appl. Psychol. 93, 498–512. 10.1037/0021-9010.93.3.498 18457483

[B67] Mitchell J. T. Everly G. S. (2000). Critical incident stress management and critical incident stress debriefings: evolutions, effects and outcomes, in Psychological Debriefing: Theory, Practice, and Evidence, eds Raphael B. Wilson J. P. (Cambridge: Cambridge University Press), 71–90.

[B68] Morina N. Wicherts J. M. Lobbrecht J. Priebe S. (2014). Remission from post-traumatic stress disorder in adults: a systematic review and meta-analysis of long term outcome studies. Clin. Psychol. Rev. 34, 249–255. 10.1016/j.cpr.2014.03.002 24681171

[B69] Mucci N. Giorgi G. Fiz Perez J. Iavicoli I. Arcangeli G. (2015). Predictors of trauma in bank employee robbery victims. Neuropsychiatr. Dis. Treat. 11, 2605–2612. 10.2147/NDT.S88836 26504393 PMC4605248

[B70] Nandi A. Galea S. Tracy M. Ahern J. Resnick H. Gershon R.. (2004). Job loss, unemployment, work stress, job satisfaction, and the persistence of post-traumatic stress disorder one year after the September 11 attacks. J. Occup. Environ. Med. 46, 1057–1064. 10.1097/01.jom.0000141663.22902.0a 15602180

[B71] Norberg A. L. Lindblad F. Boman K. K. (2006). Support-seeking, perceived support, and anxiety in mothers and fathers after children's cancer treatment. Psychooncology 15, 335–343. 10.1002/pon.960 16106491

[B72] North C. S. Tivis L. McMillen J. C. Pfefferbaum B. Cox J. Spitznagel E. L.. (2002). Coping, functioning, and adjustment of rescue workers after the Oklahoma City bombing. J. Trauma Stress 15, 171–175. 10.1023/A:1015286909111 12092908

[B73] O'Donnell M. L. Grant G. Alkemade N. Spittal M. Creamer M. Silove D.. (2015). Compensation seeking and disability after injury: the role of compensation-related stress and mental health. J. Clin. Psychiatry 76, e1000–e1005. 10.4088/JCP.14m09211 26335085

[B74] Ozer E. J. Best S. R. Lipsey T. L. Weiss D. S. (2003). Predictors of post-traumatic stress disorder and symptoms in adults: a meta-analysis. Psychol. Bull. 129, 52–71. 10.1037/0033-2909.129.1.52 12555794

[B75] Paggi M. E. Jopp D. S. (2015). Outcomes of occupational self-efficacy in older workers. Int. J. Aging Hum. Dev. 80, 357–378. 10.1177/0091415015607640 26394821 PMC4758670

[B76] Paunović N. Öst L. G. (2004). Psychometric properties of a Swedish translation of the Clinician-Administered PTSD scale-diagnostic version. J. Trauma Stress 18, 161–164. 10.1002/jts.20013 16281209

[B77] Pitman R. K. Sanders K. M. Zusman R. M. Healy A. R. Cheema F. Lasko N. B.. (2002). Pilot study of secondary prevention of posttraumatic stress disorder with propranolol. Biol. Psychiatry 51, 189–192. 10.1016/S0006-3223(01)01279-3 11822998

[B78] Prati G. Pietrantoni L. (2009). Optimism, social support, and coping strategies as factors contributing to post-traumatic growth: a meta-analysis. J. Loss Trauma 14, 364–388. 10.1080/15325020902724271

[B79] Schaefer J. A. Moos R. H. (1998). The context for post-traumatic growth: life crises, individual and social resources, and coping, in Post-Traumatic Growth: Positive Changes in the Aftermath of Crisis, eds Tedeschi R. Park C. Calhoun L. (Mahwah, NJ: Erlbaum), 99–126.

[B80] Schwartz A. C. Bradley R. L. Sexton M. Sherry A. Ressler K. J. (2005). Post-traumatic stress disorder among African Americans in an inner city mental health clinic. Psychiatry Serv. 56, 212–215. 10.1176/appi.ps.56.2.212 15703352

[B81] Schwarzer R. Knoll N. (2007). Functional roles of social support within the stress and coping process: a theoretical and empirical overview. Int. J. Psychol. 42, 243–252. 10.1080/00207590701396641

[B82] Schyns B. von Collani G. (2002). A new occupational self-efficacy scale and its relation to personality constructs and organizational variables. Eur. J. Work Organ. Psychol. 11, 219–241. 10.1080/13594320244000148

[B83] Setti I. Argentero P. (2016). Traumatization and PTSD in rescue workers: prevention, assessment, and interventions, in Comprehensive Guide to Post-Traumatic Stress Disorders eds Martin C. R. Preedy V. R. Patel V. B. (Basel: Disorder Springer International Publishing, Springer), 301–317.

[B84] Shevlin M. Adamson G. (2005). Alternative factor models and factorial invariance of the GHQ-12: a large sample analysis using confirmatory factor analysis. Psychol. Assess. 17, 231–236. 10.1037/1040-3590.17.2.231 16029110

[B85] Sica C. Magni C. Ghisi M. Altoè G. Sighinolfi C. Chiri L. R. (2008). Coping orientation to problems experienced-Nuova Versione Italiana (COPE-NVI): uno strumento per la misura degli stili di coping. Psicoterapia Cognitiva e Comportamentale 14, 27–53.

[B86] Skogstad M. Skorstad M. Lie A. Conradi H. S. Heir T. Weisæth L. (2013). Work-related post-traumatic stress disorder. Occup. Med. 63, 175–182. 10.1093/occmed/kqt003 23564090

[B87] Söndergaard H. P. (2008). The work environment, critical incidents, debriefing and psychological functioning-a study of trade union members in Sweden. Scand. J. Work Environ. Health Suppl. 6, 111–116.

[B88] Taylor S. E. Stanton A. L. (2007). Coping resources, coping processes, and mental health. Annu. Rev. Clin. Psychol. 3, 377–401. 10.1146/annurev.clinpsy.3.022806.091520 17716061

[B89] Thoits P. A. (2011). Mechanisms linking social ties and support to physical and mental health. J. Health Soc. Behav. 52, 145–161. 10.1177/0022146510395592 21673143

[B90] Thoresen S. Tambs K. Hussain A. Heir T. Johansen V. A. Bisson J. I. (2010). Brief measure of post-traumatic stress reactions: impact of event scale-6. Soc. Psychiatry Psychiatr. Epidemiol. 45, 405–412. 10.1007/s00127-009-0073-x 19479171

[B91] Tsai J. Harpaz-Rotem I. Pietrzak R. H. Southwick S. M. (2012). The role of coping, resilience, and social support in mediating the relation between PTSD and social functioning in veterans returning from Iraq and Afghanistan. Psychiatry 75, 135–149. 10.1521/psyc.2012.75.2.135 22642433

[B92] Van der Kolk B. A. (1989). The compulsion to repeat the trauma. Psychiatr. Clin. N. Am. 12, 389–411. 2664732

[B93] Van der Velden P. G. Bosmans M. W. G. van der Meulen E. Vermunt J. K. (2016). Pre-event trajectories of mental health and health-related disabilities, and post-event traumatic stress symptoms and health: a 7-wave population-based study. Psychiatry Res. 246, 466–473. 10.1016/j.psychres.2016.10.024 27792976

[B94] Van der Velden P. G. Grievink L. Kleber R. J. Drogendijk A. N. Roskam A. J. R. Marcelissen F. G.. (2006). Post-disaster mental health problems and the utilization of mental health services: a four-year longitudinal comparative study. Adm. Policy Ment. Health 33, 279–288. 10.1007/s10488-005-0027-x 16400531

[B95] Van der Velden P. G. van der Burg S. van der Steinmetz C. H. D. van den Bout J. (1992). Slachtoffers van Bankovervallen [Victim of Bank Robberies]. Houten, NL:Bohn Stafleu Van Loghem.

[B96] van der Velden P. G. Yzermans C. J. Kleber R. J. Gersons B. P. R. (2007). Correlates of mental health services utilization 18 months and almost 4 years post-disaster among adults with mental health problems. J. Trauma Stress 20, 1029–1039. 10.1002/jts.20273 18157885

[B97] Vinokur A. D. Pierce P. F. Lewandowski-Romps L. Hobfoll S. E. Galea S. (2011). Effects of war exposure on air force personnel's mental health, job burnout and other organizational related outcomes. J. Occup. Health Psychol. 16, 3–17. 10.1037/a0021617 21280941 PMC3148072

[B98] Wahlström L. Michélsen H. Schulman A. Backheden M. (2008). Different types of exposure to the 2004 tsunami are associated with different levels of psychological distress and post-traumatic stress. J. Trauma Stress 21, 463–470. 10.1002/jts.20360 18956445

[B99] Weiss D. S. Marmar C. R. (1997). The impact of event scale-revised, in Assessing Psychological Trauma and Ptsd: A Handbook for Practitioners, eds Wilson J. P. Keane T. M. (New York, NY: Guilford Press), 399–411.

[B100] Williams N. R. Davey M. Klock-Powell K. (2003). Rising from the ashes: stories of recovery, adaptation and resiliency in burn survivors. Soc. Work Health Care 36, 53–77. 10.1300/J010v36n04_04 12836780

[B101] Wisnivesky J. P. Teitelbaum S. L. Todd A. C. Boffetta P. Crane M. Crowley L.. (2011). Persistence of multiple illnesses in World Trade Center rescue and recovery workers: a cohort study. Lancet 378, 888–897. 10.1016/S0140-6736(11)61180-X 21890053 PMC9453925

[B102] Yuan K. C. Ruo Yao Z. Zhen Yu S. Xu Dong Z. Jian Zhong Y. Edwards J. G.. (2013). Prevalence and predictors of stress disorders following two earthquakes. Int. J. Soc. Psychiatry 59, 525–530. 10.1177/0020764012453233 22851134

[B103] Zatzick D. Jurkovich G. J. Rivara F. P. Wang J. Fan M. Y. Joesch J. (2008). A national US study of post-traumatic stress disorder, depression, and work and functional outcomes after hospitalization for traumatic injury. Ann. Surg. 248, 429–437. 10.1097/SLA.0b013e318185a6b8 18791363

